# Assessment of boron accumulation and pollution levels and their relation to soil classification

**DOI:** 10.1007/s10653-026-03312-7

**Published:** 2026-06-20

**Authors:** Yakup Kenan Koca, Halil Aytop, Cafer Hakan Yılmaz, Ömer Faruk Demir, Muhammet Raşit Sünbül, Hatice Mehtap Erayman, Hüseyin Dikici

**Affiliations:** 1 Department of Soil Science and Plant Nutrition, Faculty of Agriculture, Cukurova University, Adana, Türkiye; 2 Soil and Water Research Department, East Mediterranean Transitional Zone Agricultural Research of Institute (TAGEM/MoAF), Kahramanmaraş, Türkiye; 3 Department of Soil Science and Plant Nutrition, Faculty of Agriculture, Kahramanmaras Sutcu Imam University, Kahramanmaras, Türkiye

**Keywords:** Distribution map, FAO-UNESCO soil classification, Soil contamination, Risk assessment

## Abstract

Because the thresholds for Boron (B) deficiency and toxicity in soils are very close, continuous monitoring of B levels is essential for sustainable soil and environmental management. This study aimed to determine total B concentrations in agricultural soils of the Seyhan Plain (Türkiye), evaluate associated environmental, ecological, and human health risks, and investigate the relationship between B concentrations and soil classification. A total of 51 surface soil samples were collected from conventionally managed cotton fields in the Seyhan Plain. Total B concentrations were determined by ICP-OES following microwave digestion. Pollution and ecological risk assessments were conducted using the enrichment factor (EF), contamination factor (Cf), geoaccumulation index (Igeo), and ecological risk factor (Er). Non-carcinogenic human health risks were evaluated separately for children and adults. Boron concentrations were also compared among different FAO–UNESCO soil classes. The mean B concentration in the studied soils was 43.72 mg/kg, which is approximately 2.6 t higher than the Upper Continental Crust (UCC) background value. The mean EF, Igeo, Cf, and Er values were 2.39, 0.73, 2.57, and 5.14, respectively, indicating moderate enrichment, moderate contamination, and low ecological risk. Human health risk assessment revealed that boron exposure through soil does not pose significant non-carcinogenic risks to either children or adults (HI < 1). Boron concentrations varied among soil classes, with the highest mean values observed in Gleyic Solonchak soils (50.72 mg kg^−1^) and the lowest in Calcaric Fluvisols (41.96 mg kg^−1^), although these differences were not statistically significant (Kruskal–Wallis, p = 0.123). The results suggest that both geogenic factors (parent material, soil characteristics, and drainage conditions) and anthropogenic factors (irrigation and agricultural practices) may have contributed to boron accumulation in the study area. Although ecological and human health risks were low, the observed boron enrichment highlights the importance of continued monitoring and site-specific management strategies for sustainable agricultural production in the Seyhan Plain.

**Supplementary Information:**

The online version contains supplementary material available at 10.1007/s10653-026-03312-7.

## Introduction

Soils exhibit variable nutrient compositions influenced by their parent materials. Among these nutrients, boron (B) is a crucial micronutrient essential for optimal plant growth and physiological functions. Globally, the mean total B content in soils is approximately 42 mg per kg, with typical ranges spanning from 9 to 85 mg/kg (Aytop et al., 2023; Kabata-Pendias, 2010). Natural sources of B primarily include borosilicate minerals, volcanic activity, geothermal processes, groundwater, and seawater (Aytop et al., 2023; Helvacı, 2017; Varol et al., 2023). Türkiye is notably significant in global B reserves, owning approximately 73% of the world’s reserves—around 1,100,000 tonnes ranking first worldwide, followed by the United States (40,000 tonnes), Chile (35,000 tonnes), and China (24,000 tonnes) (Anonymous, 2022; Aytop et al., 2023). Despite this abundance, only a small fraction of Türkiye’s B reserves is in bioavailable forms accessible to plants (Brdar-Jokanović, 2020; Sun et al., 2019). Studies indicate that roughly half of Turkish soils contain B levels that are inadequate for optimal plant growth, highlighting a significant deficiency in bioavailable B within agricultural soils (Çakmak, 2016; Kıllıoğlu, 2022).

B is predominantly absorbed by plants in the form of boric acid. The margin between deficient, optimal, and toxic soil B concentrations is notably narrow (Aytop et al., 2023; Brdar-Jokanović, 2020; Yau & Ryan, 2008). B is required by plants in very small quantities; however, it plays essential roles in cell wall formation, membrane integrity, reproductive development, sugar transport, and various metabolic processes. B deficiency generally occurs when plant-available B concentrations fall below 0.5 mg kg^−1^, whereas toxicity symptoms may appear when available B exceeds approximately 5 mg kg^−1^, depending on plant species and soil conditions. Because the range between deficiency and toxicity is relatively narrow, B is considered one of the most critical micronutrients in crop production (Aytop et al., 2023; Varol et al., 2023). Although total B concentrations in soils may be high, only a small fraction is present in plant-available forms. Soil properties such as pH, texture, clay mineralogy, organic matter content, and moisture conditions strongly influence B availability and uptake by plants (Brdar-Jokanović, 2020). Consequently, elevated soil B levels do not invariably result in phytotoxicity; such effects are often influenced by soil characteristics, lime content, and the specific plant species. Particularly in arid and semi-arid regions, high B concentrations, compounded by irrigation water quality, can detrimentally impact crop productivity. A study in the Euphrates Basin (Özbek, 2004) reported irrigation water B levels reaching up to 0.52 mg/kg, which contributed to a decline in water quality.

Factors such as B concentration in soils; clay mineral type (Su & Suarez, 1995); organic matter content (Havlin et al., 2005); soil pH, texture, temperature, and moisture level (Xu et al., 2001) significantly influence soil properties (Cüre, 2023). Under acidic to neutral conditions, boron mainly occurs as boric acid (H_3_BO_3_), which is relatively mobile in the soil solution. As soil pH increases, boron is progressively converted to borate ions B(OH)^−^_4_, which can be more strongly adsorbed by clay minerals, iron and aluminum oxides, and carbonate surfaces, thereby reducing its mobility and plant availability. Soil organic matter also plays an important role in boron retention through adsorption and complexation processes. Consequently, soils with higher clay and organic matter contents generally exhibit greater boron retention capacities, whereas coarse-textured soils are more susceptible to boron leaching (Bolan et al., 2023; Brdar-Jokanović, 2020; Goldberg, 1997).

Soil classification describes soils based on pedogenic properties that affect their formation, parent material, drainage, horizon development, and mineralogical composition. As a result, B element accumulation, mobility, and availability to plants differ among soils categorized into various classes. For instance, clay-rich soils classified as Vertisols can adsorb more B, whereas well-drained young soils, such as Fluvisols, may experience B loss through leaching. Therefore, soil classification should be regarded not only as a morphological criterion but also as a crucial factor in nutrient management. Moreover, evaluating boron concentrations according to soil classification may help identify soils that are more susceptible to boron accumulation, toxicity, or deficiency and may contribute to the development of soil-specific boron management strategies.

Soil ecosystems experience notable shifts in nutrient element balances due to human activities such as agriculture, industrial emissions, and mining, alongside natural sources like parent material. In addition, conventional agricultural practices may contribute to boron accumulation in soils through the repeated application of synthetic fertilizers, micronutrient formulations, irrigation water, and agrochemicals. Although boron fertilization is often required to correct deficiencies, excessive or unbalanced applications may increase soil boron concentrations and alter nutrient interactions within the soil–plant system (Aytop et al., 2023; Varol et al., 2023). Elevated boron levels can interfere with the uptake and utilization of other nutrients, potentially leading to nutritional imbalances and reduced crop performance (Bolan et al., 2023; Brdar-Jokanović, 2020). This can lead to the buildup of trace elements such as B, potentially disrupting ecological equilibrium and posing health risks to humans.

Consequently, evaluating element accumulation in soils requires considering not only elemental concentrations but also their environmental, ecological, and human health implications. Various pollution indices, including the enrichment factor (EF), contamination factor (Cf), geoaccumulation index (Igeo), and ecological risk factor (Er), are widely used to assess contamination levels, identify potential sources, and estimate ecological risks associated with trace element accumulation. High concentrations of B in soil or irrigation water may be transferred to humans through the food chain and can potentially cause adverse health effects (Bolan et al., 2023; Uluisik et al., 2018).

Although boron contamination has been investigated in several studies, comprehensive assessments integrating total boron concentrations, ecological risk, and human health risk remain limited. One important reason is that boron received relatively little attention in ecological risk assessment for many years because a toxicity coefficient required for the calculation of ecological risk indices was not available. Xu et al. (2020) proposed a boron toxicity coefficient and demonstrated its applicability in ecological risk assessment studies. Despite this development, studies simultaneously evaluating total boron accumulation, ecological risk, human health risk, and soil classification relationships remain scarce. In Türkiye, only two studies have comprehensively assessed total boron concentrations together with ecological and human health risks in agricultural soils (Aytop et al., 2023; Varol et al., 2023). Furthermore, information regarding the relationship between boron accumulation and soil classification remains very limited. Therefore, a comprehensive assessment of boron distribution, contamination status, ecological risk, human health risk, and soil classification relationships is needed to improve our understanding of boron behavior and support sustainable soil management in the Seyhan Plain, one of the most important agricultural regions of Türkiye.

The objective of this research is to evaluate the B concentrations in soils of the Seyhan Plain, situated within the Çukurova Plain, one of Türkiye’s most significant agricultural zones known for multiple cropping cycles annually. The study further seeks to examine the correlation between B concentrations and soils categorized according to the FAO UNESCO (1974) soil classification system. Additionally, using established indices and factors such as EF, Cf, Igeo, and Er, the research assesses potential human health risks for adults and minors based on the collected data.

## Materials and methods

### Study area

**Fig. 1:**
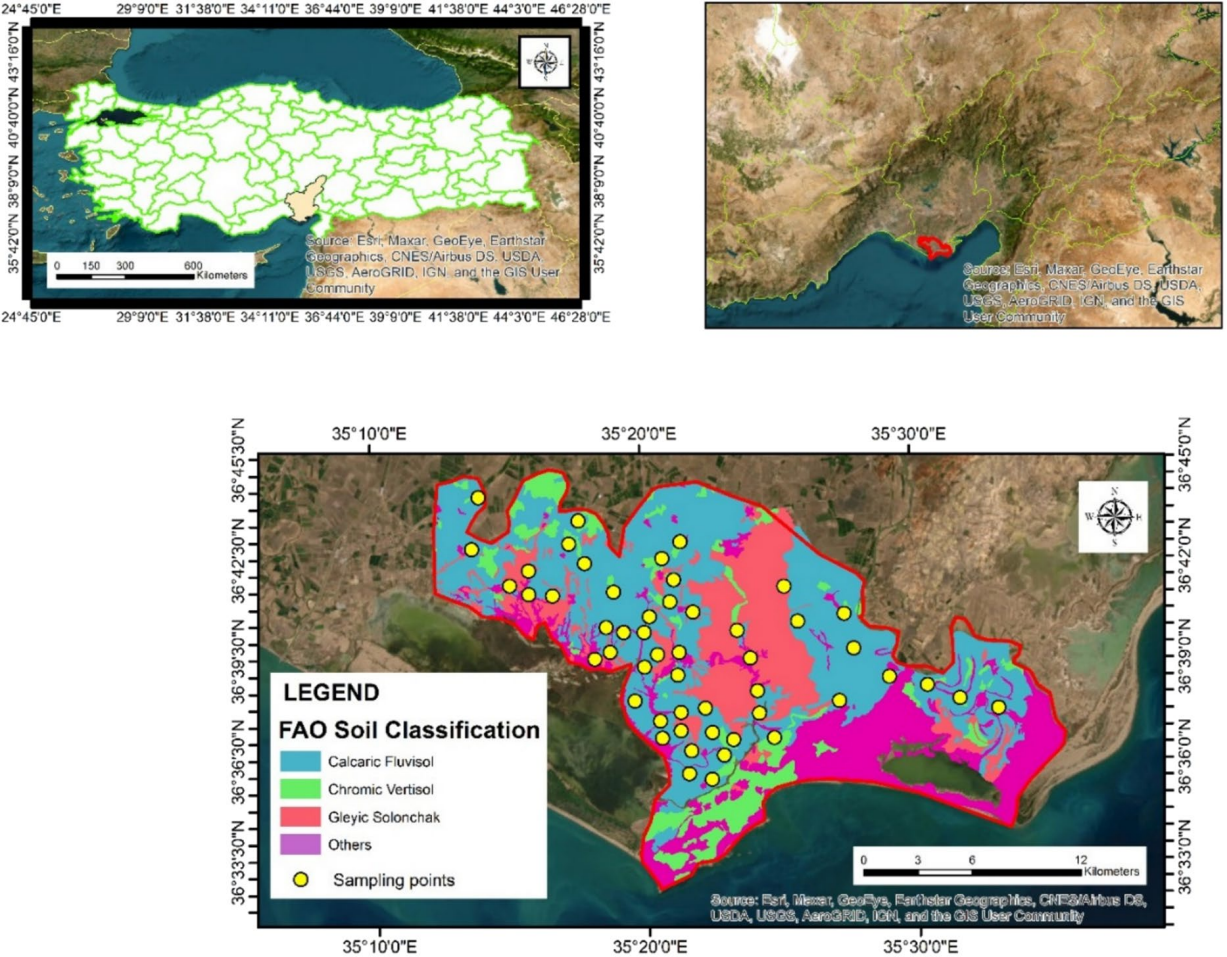
Study area location map

The research was carried out within the boundaries of Karataş district in Adana province, located in southern Türkiye. Geographically, the area is situated between 36° 04′ 5″ N, 35° 01′ 2″ E and 36° 03′ 5″ N, 35° 03′ 4″ E (Fig. 1). The Akyatan and Ayyayan Lagoons, which are among the region’s most significant water sources, are immediately adjacent to the sampling site. Intensive agricultural activities are carried out throughout the entire working area. The climate of Adana province is classified as Mediterranean, with a mean annual temperature of 19.3 ℃ and a mean annual precipitation of 667.5 mm. Summer temperatures can reach notably high levels, up to 45.7 ℃ (Anonymous, 2025). Rainfall occurs predominantly during the winter months.

A detailed soil survey and mapping of the study area were conducted by Dinç et al. (1990). It has been identified that the Arpacı, Arıklı, Çanakçı, Helvacı, and Oymaklı series are prevalent throughout the area. Among these, the Arpacı, Çanakçı, and Oymaklı series are relatively young soils classified as Calcaric Fluvisol. The soils of the middle-aged Helvacı series are classified as Gleyic Solonchak, while the Arıklı series soils, which are somewhat older than the others, are classified as Chromic Vertisol (FAO UNESCO, 1974). The Arpacı series is affected by groundwater, and salinity in the Helvacı series partially impedes agricultural productivity.

### Soil sampling, laboratory analyses and spatial distribution studies

A total of fifty-one surface soil samples were collected from agricultural fields cultivated with cotton under conventional farming practices. Sampling locations were selected to represent the major agricultural soils of the study area, and no specific preference was given to sites located near potential boron sources. The sampling design aimed to provide a representative assessment of boron concentrations across the cotton-growing areas of the Seyhan Plain. The sampling points were distributed among the major soil series of the study area, including Arpacı (n = 19), Oymaklı (n = 15), Çanakçı (n = 6), Helvacı (n = 8), and Arıklı (n = 3). The Global Positioning System (GPS) was used to determine the coordinates of each sampling location. The sampling locations were not based on the LUCAS (Land Use/Cover Area Frame Survey) network. Soil samples collected from a depth of 0–20 cm using a steel shovel were transported in nylon bags (Aytop et al., 2023; Yılmaz, 2023). After air drying, the samples were sieved through 2- and 0.5-mm sieves, and approximately 0.3 g of each sample was digested using a microwave digestion system with a mixture of 6 mL HNO_3_ and 5 mL HCl. The digested solutions were filtered through filter paper and diluted to a final volume of 50 mL with ultrapure water. Concentrations of B, Al, and Fe were then determined using ICP-OES. All reagents used were of analytical grade (Merck, Germany). Organic Matter (%) was quantified utilizing the modified Walkley–Black method (Jackson, 1979). Lime content was measured with a Scheibler calcimeter (Soil Survey Lab Staff, 1992). Additionally, soil pH and electrical conductivity (EC) were determined from saturated soil paste extracts according to standard soil analysis procedures. Prior to use, all glassware and plasticware were soaked in 3% HNO_3_ for 24 h and rinsed thoroughly with ultrapure water. Quality control and assurance procedures including reagent blanks, spiked samples, and triplicate analyses were performed to ensure the reliability of the analytical results. Method performance was verified through the analysis of the EnviroMAT SS-2 certified reference material, in which 8.3 mg kg^−1^ of B was measured against the certified value of 8.5 mg kg^−1^, corresponding to a 97.7% recovery. The method showed good sensitivity, with a limit of detection (LOD) of 0.005 mg kg^−1^ and a limit of quantification (LOQ) of 0.017 mg kg^−1^. ArcGIS 10.4 (ESRI, Redlands, CA, USA) was used for spatial analysis. The Inverse Distance Weighting (IDW) interpolation technique was employed to estimate boron concentrations at unsampled locations and to produce the spatial distribution map of boron in the study area.

### Pollution status and ecological risk assessment

Soil contamination is generally assessed using contamination indices (Akbay et al., 2023; Taşpınar et al., 2022; Varol et al., 2020). In this study, enrichment factor (EF), geoaccumulation index (Igeo), and contamination factor (Cf) were applied to evaluate boron (B) contamination, while the potential ecological risk factor (Er) was used to estimate ecological risk for using B. Iron (Fe) was selected as the reference element for EF (Ateş et al., 2023), and upper continental crust (UCC) values were used as background concentrations due to the absence of local B background data (Rudnick & Gao, 2003). The equations, explanations, and pollution classes for the EF, Igeo, Cf, and Er indices are given in Tables S1 and S2.

### Health risk assessment studies

Health risk assessment serves as a widely used framework for appraising potential human health impacts due to exposure to environmental contaminants. In this study, a comprehensive risk assessment was conducted to evaluate the risks faced by adults and children living in the research area who may be exposed to B present in soils via ingestion, inhalation, or dermal contact (USEPA, 2023). The parameters employed for assessing non-carcinogenic effects, including hazard quotients, along with detailed descriptions of the relevant formulas, are provided in Table S2.

## Results

### Boron concentrations in soils and environmental-ecological risks

The concentration of boron (B) in the soils within the study area varies from 17.89 to 72.83 mg/kg. The mean total contents of B and iron (Fe) were calculated to be 43.72 mg/kg and 41,818 mg/kg, respectively (Table 1). These concentrations were further classified according to soil series and their associated basic groups. The study encompasses five soil series, which are distributed among three groups. Notably, the Helvacı series (Gleyic Solonchak) exhibited the highest mean B concentration at 50.72 mg.kg^−1^, followed by the Arıklı series (Chromic Vertisol) with 48.52 mg.kg^−1^, and the Arpacı-Çanakcı-Ovmaklı series (Calcaric Fluvisol) with 41.96 mg.kg^−1^. In the study, Fe served as the reference element for the calculation of pollution indices. Fe concentrations ranged from 29,119 to 59,382 mg kg^−1^. The coefficient of variation (CV) was 24.51%, while the skewness value was found to be 0.50. The mean pH value of the soil was measured at 7.56, indicating a slightly alkaline condition. The electrical conductivity (EC) was found to be 0.96 dS/m, which suggests that the soil is non-saline. Furthermore, the organic matter content was determined to be 2.02%, indicating that the soil is classified as having a medium level of organic matter.

**Table 1:** Boron content in soils of study area and other research sites

	B (mg kg^−1^)	Fe (mg kg^−1^)	pH	EC (dS/m)	OM (%)	CaCO_3_(%)	EF	Igeo	Cf	Er
Mean	43.72	41,818	7.56	0.96	2.02	20.68	2.39	0.73	2.57	5.14
Maximum	72.83	59,382	8.05	2.56	2.88	36.41	3.45	1.51	4.28	8.57
Minimum	17.89	29,119	6.56	0.13	1.02	12.99	1.38	0.51	1.05	2.10
Coefficient of variation (%)	24.5	–	–	–	–	–	–	–	–	–
Skewness	0.50					–				
Upper continental crust (UCC), Rudnick and Gao (2003)	17	–	–	–	–	–	–	–	–	–
World soil average (WSA), Kabata-Pendias (2010)	42	–	–	–	–	–	–	–	–	–
Agricultural soils (USA), Kabata-Pendias (2010)	33	–	–	–	–	–	–	–	–	–
Amik Plain (Türkiye), Aytop et al. (2023)	29	–	–	–	–	–	2.56	0.17	1.73	3.46
Agricultural soils (İzmir- Türkiye), Varol ey al. (2023)	47	–	–	–	–	–	9.82	0.85	2.77	5.54
Continental-scale distribution and source identification of boron in soils of China (2025)	50.1	–	–	–	–	–	–	–	–	–

A strong positive correlation was observed between Fe and Al concentrations (r = 0.882), suggesting a common geogenic origin and supporting the use of Fe as a reference element in the EF calculations. According to the results of the environmental risk analysis of the study area, the mean EF value of the soils was determined to be 2.39. The mean Igeo and Cf values were calculated as 0.73 and 2.57, respectively. Based on these environmental index results, the study area has been classified as moderately enriched (EF), unpolluted to moderately polluted (Igeo) and moderately contaminated (Cf) in terms of B contamination. The Er result caused by B in the area was found to be 5.14, and as this value is below 40, it has been classified as posing a low potential ecological risk.

When analyzing soil B content by soil series, the highest average B concentration was observed in the Gleyic solonchak series at 50.72 mg.kg^−1^ (Table 1). This was followed by the Chromic Vertisol series with an average of 48.52 mg.kg^−1^, and the Calcaric Fluvisol series at 41.96 mg.kg^−1^. The Fe content of the soils was measured, with an average of 43,246 mg.kg^−1^ in the series classified as Gleyic Solonchak according to FAO-UNESCO. Additionally, the Fe content was 46,951 mg.kg^−1^ in the Chromic Vertisol series and 41,147 mg.kg^−1^in the Calcaric Fluvisol series (Table 2).

**Table 2:** Selected soil properties and boron concentrations according to FAO–UNESCO soil classes (1974)

Soil Class	n	B (mg kg^−1^)	Fe (mg kg^−1^)	pH	EC (dS/m^−1^)	OM (%)	Lime (%)
Calcaric Fluvisol	40	41.96 ± 10.30	41,147 ± 5145	7.56 ± 0.24	0.96 ± 0.60	2.02 ± 0.44	20.72 ± 4.42
Chromic Vertisol	3	48.52 ± 10.48	46,951 ± 10,789	7.47 ± 0.28	1.47 ± 0.56	2.04 ± 0.15	20.11 ± 3.41
Gleyic Solonchak	8	50.72 ± 11.42	43,246 ± 4300	7.75 ± 0.25	1.33 ± 0.94	1.94 ± 0.53	20.73 ± 3.83

Values are presented as mean ± standard deviation. The Kruskal–Wallis test indicated no statistically significant differences in total boron concentrations among soil classes (p = 0.123)

**Fig. 2:**
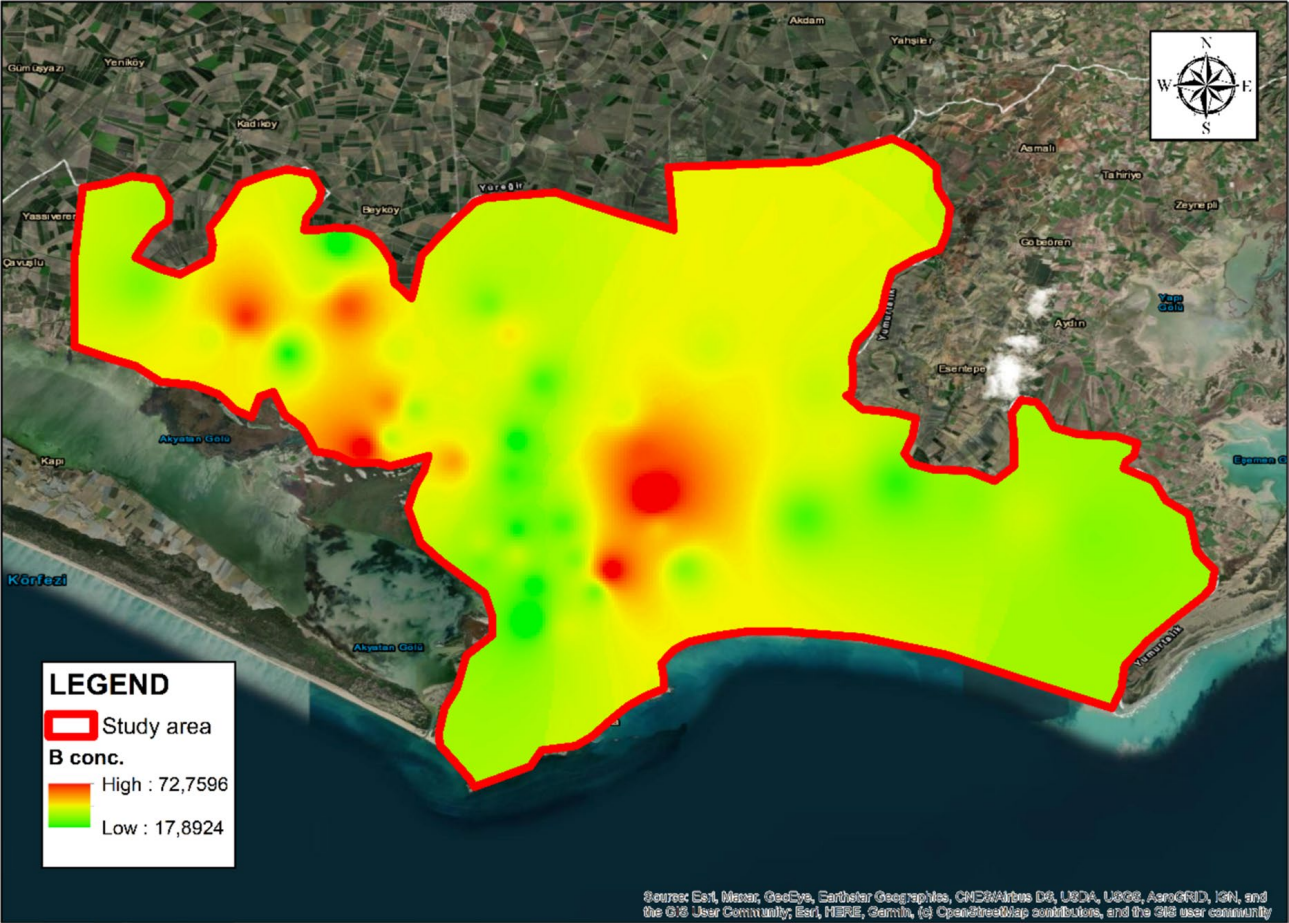
Distribution map of boron content in the study area

B concentrations are reported to be elevated in regions characterized by soils classified as Gleyic Solonchak, predominantly located in the central and western sectors of the study area. Conversely, areas at the western and southern extremities exhibit lower B levels (Fig. 2). The dominant soil series in these regions are Chromic Vertisol and Calcaric Fluvisol.

### Health risk assessment

The assessed non-carcinogenic health risk levels for agricultural soils in the study area are below the threshold value of 1 (Table 3). This suggests that B exposure does not pose significant non-carcinogenic health risks to children or adults.

**Table 3:** Non-carcinogenic health risk results (HQ and HI) calculated for adults and children in the study area

	Health risks for children				Health risks for adults			
	HQ_ing_	HQ_inh_	HQ_der_	HI	HQ_ing_	HQ_inh_	HQ_der_	HI
Mean	6.99E−04	1.54E−05	6.64E−06	7.21E−04	5.82E−05	1.54E−05	1.23E−06	1.54E-05
Maximum	1.16E−03	2.57E−05	1.10E−05	1.20E−03	9.70E−05	2.57E−05	2.05E−06	2.57E−05
Minimum	2.86E−04	6.31E−06	2.71E−06	2.95E−04	2.38E−05	6.31E−06	5.03E−07	6.31E−06

## Discussion

### Boron levels in soil and influencing factors

The B content in the agricultural soils of the study area exceeds the levels found in the Upper Continental Crust (UCC), the World Soil Average (WSA), and the typical values for American agricultural soils. The mean B concentration was also comparable to that reported for soils of China (50.1 mg kg^−1^), where parent material, soil properties, and land-use practices were identified as important factors controlling boron distribution (Liu et al., 2025). When compared to regional studies in Türkiye, the B concentration is approximately 1.5 t higher than that of soils in the Amik Plain, yet slightly lower than in olive-growing regions of Izmir. Additionally, numerous studies have demonstrated that agricultural practices such as fertilization and pesticide application significantly contribute to increased soil B levels (Aytop et al., 2023; Varol et al., 2023). Elevated B concentrations have also been reported in regions where groundwater irrigation is prevalent; however, data on groundwater B levels in the current study area are lacking (Fatnassi et al., 2025; Hosseinifard et al., 2015; Shouse et al., 2006). Therefore, both geogenic and anthropogenic factors may have influenced the boron concentrations observed in the study area. The coefficient of variation (CV) provides insight into the pollution source. A CV below 15% generally indicates that natural sources-such as parent material-are the primary contributors, with minimal human influence (Chen, 2023; Li et al., 2022; Varol et al., 2023). In the study area, the CV was 24.5% and the skewness was 0.50 (less than 1). These metrics suggest a moderate variability in pollutant levels, implying that both natural processes and human activities may contribute to pollution in the region. The repeated application of fertilizers, micronutrient amendments, and irrigation water in conventionally managed cotton fields may contribute to boron accumulation over time. However, detailed records regarding irrigation water quality, fertilizer application rates, and agrochemical use were not available for the sampled fields; therefore, their individual contributions could not be quantitatively evaluated in the present study.

According to Pearson correlation analysis, there was no significant linear relationship (p > 0.05) between total soil B, organic matter (%), pH, electrical conductivity (EC), and lime content. All correlation coefficients (r) were below ± 0.22, indicating weak relationships among these variables. Correlation analysis revealed that total boron concentrations were not significantly correlated with soil pH (r = 0.001, p = 0.995), EC (r = −0.188, p = 0.185), organic matter content (r = 0.220, p = 0.120), or lime content (r = -0.129, p = 0.365). These findings indicate that the variability of total boron concentrations in the study area cannot be explained solely by these soil properties. Although soil pH, EC, organic matter, and lime content are known to influence boron behavior and availability in soils, the present study did not reveal statistically significant relationships between these properties and total boron concentrations. Therefore, these factors cannot be considered the primary controls of total boron variability in the study area.

The spatial distribution map revealed a distinct boron hotspot in the central and western parts of the study area. This hotspot largely coincides with the occurrence of Gleyic Solonchak soils, which are characterized by poor drainage conditions, shallow groundwater influence, and the accumulation of soluble salts. Under these conditions, boron may accumulate in the surface horizons due to limited leaching and the upward movement of dissolved salts through capillary rise. In addition, the proximity of the study area to coastal environments and lagoon systems may contribute to the enrichment of soluble elements, including boron. The long-term use of irrigation water in conventionally managed cotton fields may further enhance boron accumulation, particularly in areas where drainage is restricted. Furthermore, the relatively high clay content commonly associated with these soils may increase boron retention through adsorption processes, reducing boron mobility and promoting its accumulation in the soil profile. Therefore, the observed hotspot is likely the result of the combined effects of soil properties, hydrological conditions, and long-term agricultural management practices rather than a single source of boron enrichment (Bolan et al., 2023; Goldberg, 1997; Shouse et al., 2006). Since all soil samples were collected from conventionally managed cotton fields, agricultural practices may also have contributed to the observed boron distribution patterns. The repeated application of fertilizers, micronutrient amendments, and irrigation water over long periods can influence boron accumulation in agricultural soils. Although boron deficiency is frequently corrected through fertilization, excessive or unbalanced applications may increase boron concentrations in the soil. Therefore, in addition to natural factors such as soil properties and drainage conditions, long-term agricultural management practices may have affected boron accumulation in the study area (Aytop et al., 2023; Brdar-Jokanović, 2020; Varol et al., 2023).

### Soil contamination status

The EF value for the study area was 2.39, indicating a ‘Moderate enrichment’ level, which is lower than the values reported in two previous Turkish studies. In contrast, soils in İzmir demonstrated a ‘Significant enrichment’ with an EF of 9.82 (Table 1). This discrepancy is likely attributable to İzmir’s proximity to Türkiye’s B deposits. Furthermore, the selection of reference and background values in the EF calculation can markedly affect enrichment evaluations (Aytop et al., 2023). The Igeo value for the study area is below 1, classifying it as “Unpolluted to moderately polluted.” Similarly, the Igeo values for the Amik Plain and İzmir agricultural soils also fall below 1, corroborating the findings of this study. Regarding contamination factor (Cf), the study area was categorized as “Moderate contamination” with a value of 2.57. When comparing these Cf results to other Turkish studies, soils in two regions exhibited a similar classification as “Moderate contamination”. Since the mean Er result for the total B content in the work area is below 40, it suggests that there is no significant ecological risk.

It should be noted that the Upper Continental Crust (UCC) values used in this study represent a widely accepted geochemical reference and may not fully reflect the local geogenic background of the Seyhan Plain. The importance of using regional background values derived from parent materials or deep soil horizons for contamination assessments has been highlighted in previous studies (Aytop et al., 2023). However, no regional boron background values based on deep soil horizons or uncontaminated parent materials are currently available for the study area. Therefore, UCC values were adopted to facilitate comparison with previous studies and to ensure consistency with widely applied contamination assessment approaches. Future investigations should focus on establishing local boron background concentrations to improve the accuracy of contamination assessments in the Seyhan Plain.

### Human health risk assessment caused by boron

In this research, the potential impacts of B present in soil on human health were examined separately for children and adults. The mean values of the hazard quotient for ingestion, inhalation, and dermal contact, as well as the cumulative hazard index (HI), were all found to be well below the threshold of 1 for both age groups. This suggests that B exposure levels are within safe limits for both children and adults. The mean overall risk index calculated for children (HI = 7.21 × 10^−4^) was notably higher than that for adults (HI = 1.54 × 10^−5^). This difference is attributable to children’s lower body weight and comparatively higher intake rates (EFSA, 2013; USEPA, 1989a, 1989b). Nonetheless, since all HI values were below 1 across the studied population, no indication of systemic toxicity or chronic health effects due to B exposure is expected. The primary exposure pathway identified was ingestion, with inhalation and dermal absorption contributing minimally to overall risk. These findings are consistent with similar research in the field. For instance, risk evaluations conducted by Aytop et al. (2023) and Varol et al. (2023) also identified ingestion as the main route of B exposure.

### The relationship between FAO–UNESCO (1974) soil classes and boron content

When examining the classified soils Chromic Vertisol (48.52 mg/kg B), Calcaric Fluvisol (41.96 mg/kg B), and Gleyic Solonchak (50.72 mg/kg B), differences in mean boron concentrations were observed among soil classes. As shown in Table 2, Gleyic Solonchak soils exhibited the highest mean boron concentration (50.72 mg kg^−1^) together with relatively high EC values (1.33 dS m^−1^), which may be related to the relatively more saline conditions generally associated with this soil class. Chromic Vertisols contained 48.52 mg kg^−1^ boron and were characterized by the highest Fe concentrations (46,951 mg kg^−1^). In contrast, Calcaric Fluvisols exhibited the lowest mean boron concentration (41.96 mg kg^−1^) and lower EC values. Although the differences in boron concentrations among soil classes were not statistically significant (Kruskal–Wallis, p = 0.123), these variations suggest that soil-specific properties may influence boron accumulation patterns. The relatively higher boron concentrations observed in Gleyic Solonchak soils may be associated with salinity, periodic water saturation, and restricted drainage conditions (Landi et al., 2019; Zhao et al., 2024). Conversely, the lower boron concentrations observed in Calcaric Fluvisols may reflect greater susceptibility to leaching processes associated with their alluvial nature and relatively efficient drainage. The moderate to high boron concentrations observed in Vertisols may be related to the clay-rich characteristics typically associated with this soil class and its retention capacity. Given that only a fraction of soil B is accessible for plant uptake, soil physicochemical properties such as clay content, pH, organic matter, and oxide compounds play crucial roles in determining B bioavailability and potential toxicity (Das & Purkait, 2020; Padbhushan & Kumar, 2017). Consequently, differences in total boron concentrations among soil classes may have implications for boron availability and management practices. Therefore, understanding soil classification systems and their associated physicochemical attributes is essential for developing effective boron management strategies.

## Conclusions

The present study evaluated total boron concentrations in agricultural soils of the Seyhan Plain and assessed their environmental, ecological, and human health implications. The results indicated that the study area was characterized by moderate boron contamination according to the enrichment factor (EF) and contamination factor (Cf), while the geoaccumulation index (Igeo) classified the soils as unpolluted to moderately polluted. The ecological risk factor (Er) revealed a low ecological risk level. Human health risk assessment showed that boron exposure through soil does not pose significant non-carcinogenic risks to either children or adults.

No significant relationships were observed between total boron concentrations and soil pH, electrical conductivity (EC), organic matter content, or lime content. The results suggest that both geogenic factors, such as parent material and soil characteristics, and anthropogenic factors, including irrigation and agricultural practices, may have contributed to boron accumulation in the study area.

Higher mean boron concentrations were observed in Gleyic Solonchak and Chromic Vertisol soils than in Calcaric Fluvisols. However, these differences were not statistically significant according to the Kruskal–Wallis test (p = 0.123). Nevertheless, the observed variations among soil classes indicate that soil classification may provide useful information for understanding boron distribution and developing site-specific boron management strategies.

Future studies should include analyses of parent materials, irrigation water, and regional background values to better identify the sources of boron and improve contamination assessments in the Seyhan Plain.

## Supplementary Information

Below is the link to the electronic supplementary material.

Supplementary file1 (DOCX 24 KB)

## Data Availability

Data is available upon reasonable request.
