# Supplementary material for: Assessment of boron accumulation and pollution levels and their relation to soil classification
Source: Environ Geochem Health. 2026 Jun 20;48(9):416. doi: 10.1007/s10653-026-03312-7 (PMC13283174; doi:10.1007/s10653-026-03312-7)
Supplement: Supplementary file 1 — Supplementary file1 (DOCX 24 KB) [file 10653_2026_3312_MOESM1_ESM.docx]

**Assessment of Boron Accumulation and Pollution Levels and Their Relation to Soil Classification**

**Affiliations**

Yakup Kenan Koca^1*^, Halil Aytop^2^, Cafer Hakan Yılmaz^2^, Ömer Faruk Demir^3^, Muhammet Raşit Sünbül^2^, Hatice Mehtap Erayman^2^, Hüseyin Dikici^3^

^1^ Department of Soil Science and Plant Nutrition, Faculty of Agriculture, Cukurova University, Adana, Türkiye

**^2^** East Mediterranean Transitional Zone Agricultural Research of Institute (TAGEM/MoAF), Soil and Water Research Department, Kahramanmaraş, Türkiye

^3^ Department of Soil Science and Plant Nutrition, Faculty of Agriculture, Kahramanmaras Sutcu Imam University, Kahramanmaras, Turkiye

*****Corresponding Author, E-mail: [ykkoca@cu.edu.tr](mailto:ykkoca@cu.edu.tr)

**Yakup Kenan KOCA OrcID:** 0000-0001-9285-1416

**Tables:**

Table S1. Pollution indices and ecological risk index utilised in this study

| Indices and their equalities | Explanation | Pollution class values |
| --- | --- | --- |
| $EF (Enrichment Factor)=\frac{\left( \frac{\mathrm{Cn}}{\mathrm{Cref}} \right)sample}{\left( \frac{\mathrm{Ci}}{\mathrm{Cref}} \right)background}$ | Cn represents the content of element (n), while Cref denotes the reference content of iron (Fe) used for geochemical normalization (Aytop et al., 2023) | *EF < 2 (Minimal enrichment)  *2 ≤ EF < 5 (Moderate enrichment)  *5 ≤ EF < 20 (Significant enrichment)  *20 ≤ EF < 40 (Very high enrichment)  *EF ≥ 40 (Extremely high enrichment) |
| $Igeo (Geoaccumulation index)=log2[\frac{Ci}{1.5*Bi}]$ | Bn is the background value of element (i); Cn is the content of element (n) (Müller, 1981). | *Igeo ≤ 0 (Unpolluted)  *0 < Igeo < 1 (Unpolluted to moderately polluted)  *1 < Igeo < 2 (Moderately polluted)  *2 < Igeo < 3 (Moderately to heavily polluted)  *3 < Igeo < 4 (Heavily polluted)  *4 < Igeo < 5 (Heavily to extremely polluted)  *Igeo ≥ 5 (Extremely polluted) |
| Cf (Contamination factor) = C^i^/C^i^_n_ | C^i^_n_ represents the pre-industrial baseline value of element (i), while Ci denotes the current concentration of element (i) (Hakanson, 1980). | *CF < 1 (Low contamination)  *1 < CF < 3 (Moderate contamination)  *3 < CF < 6 (Considerable contamination)  *CF ≥ 6 (Very high contamination) |
| Er (Ecological risk factor)= T^i^_r_ * C^i^_f_ | T^i^_r_ is the toxicity response coefficient of boron, with a value of 2. C^i^_f_ represents the contamination factor of boron (Hakanson, 1980; Aytop et al., 2023). | Er < 40 (Low potential ecological risk)  40 ≤ Er < 80 (Moderate potential ecological risk)  80 ≤ Er < 160 (Considerable potential ecological risk)  160 ≤ Er < 320 (High potential ecological risk)  Er ≥ 320 (Very high potential ecological risk) |

Table S2. Equations used in human risk assessments

|  | Equations | References |
| --- | --- | --- |
| Hazard quotient (HQ) | HQ_ingestion_= (C_s_ * IRS * RBA * EF * ED) / (BW * AT * RfDo * 10^6^) | USEPA (2023a), USEPA, (2023b) |
|  | HQ_dermal_= (C_s_ * SA * AF * ABS_d_ * EF * ED) / (BW * AT * RfDo * GIABS * 10^6^) |  |
|  | HQ_inhalation_ = (C_s_ * EF * ED) / (AT * RfC * PEF) |  |
|  | HI = HQ_ingestion_ + HQ_dermal_ + HQ_inhalation_ |  |

Table S3. Parameters used for health risk assessment in soil.

| Parameters and Symbols | Values and Units | References |
| --- | --- | --- |
| Averaging time - AT | 365 * LT = 25550 (carcinogenic) - days | Site specific |
| Averaging time (child) - ATc | 365 * EDc (non-carcinogenic) - days | USEPA (1989) |
| Averaging time (adult) - ATa | 365 * EDa (non-carcinogenic) - days | USEPA (1989) |
| Body weight (adult) - BWs | 72 - kg | Site specific |
| Body weight (child) - BWc | 15 - kg | USEPA (1991) |
| Element concentration - Cs | mg/kg | Site specific |
| Exposure duration (child) - Edc | 6 - years | USEPA (1991) |
| Exposure duration (adult) - Eda | 20 - years | USEPA (2023c) |
| Exposure frequency - EF | 350 - days/year | USEPA (1991) |
| Life time - LT | 70 - years | Site specific |
| Skin surface area (child) - SAc | 2373 – cm^2^ | USEPA (2011) |
| Skin surface area (adult) - SAa | 6032 – cm^2^ | USEPA (2011) |
| Soil intake ratio (adult) - IRSa | 20 – mg/day | Jia et al. (2018) |
| Soil intake ratio (child) - IRSc | 50 – mg/day | Jia et al. (2018) |
| Skin adherence factor (adult) - AFa | 0.07 – mg/cm^2^ | USEPA (2002) |
| Skin adherence factor (child) - AFc | 0.2 – mg/cm^2^ | USEPA (2002) |
| Soil ingestion ratio - IFS | Age-adjusted (8944.444) - mg/kg | USEPA (2023c) |
| Soil dermal contact factor - DFS | Age-adjusted (107495.111) - mg/kg | USEPA (2023c) |
| Relative bioavailability factor - RBA | 1 - unitless | USEPA (2023d) |
| Dermal absorption fraction - ABSd | 0.001 - unitless | USEPA (2004) |
| Oral reference dose - RfDo | 0.2 – mg/kg-day | USEPA (2023d) |
| Gastrointestinal Absorption - GIABS | 1 - unitless | USEPA (2023d) |
| Inhalation reference concentration - RFC | 0.002 – mg/m^3^ | USEPA (2023d) |
| Particulate emission factor -PEF | 1.36 * 109 – m^3^/kg | USEPA (2023d) |

References

Aytop, H., Ateş, Ö., Dengiz, O., Yılmaz, C. H., & Demir, Ö. F., 2023. Environmental, ecological and health risks of boron in agricultural soils of Amik Plain under Mediterranean conditions. Stoch. Environ. Res. Risk Assess. 37 (6), 2069–2081.

Hakanson, L., 1980. An ecological risk index for aquatic pollution control. A sedimentological approach. Water Res. 14 (8), 975–1001.

Müller, G., 1981. Die Schwermetallbelastung der sedimente des Neckars und seiner Nebenflüsse: eine Bestandsaufnahme. Chemiker- tg 105, 157–164.

USEPA, 1989. Risk Assessment Guidance for Superfund. Volume I: Human Health Evaluation Manual (Part A). Interim Final. Office of Emergency and Remedial Response. EPA/540/1-89/002.

USEPA, 1991. Human Health Evaluation Manual, Supplemental Guidance: "Standard Default Exposure Factors ". OSWER Directive 9285.6-03.

USEPA, 2002. Supplemental guidance for developing soil screening levels for superfund sites. OSWER 9355, 4–24.

USEPA, 2004. Risk Assessment Guidance for Superfund Volume I: Human Health Evaluation Manual (Part E, Supplemental Guidance for Dermal Risk Assessment) Final. OSWER 9285.7-02EP. July 2004.

USEPA, 2011. Exposure Factors Handbook 2011 Edition. National Center for Environmental Assessment, Office of Research and evelopment, Washington D.C.

USEPA, 2023a. Exposure Assessment Tools by Media - Soil and Dust. https://www.epa. gov/expobox/exposure-assessment-tools-media- oil-and-dust.

USEPA, 2023b. Regional Screening Levels (RSLs) – Equations. https://www.epa.gov/r isk/regional-screening-levels-rsls-equations.

USEPA, 2023c. Regional Screening Levels (RSLs) - User’s Guide. https://www.epa.gov/ risk/regional-screening-levels-rsls-users-guide.

USEPA, 2023d. Regional Screening Level (RSL) Summary Table (TR=1E-06 THQ=1.0). https://semspub.epa.gov/work/HQ/197414.pdf.
